# Preferences for Receiving and Sharing ADRD Related Health Information Following Biomarker Testing Among Cognitively Symptomatic Individuals and their Care Partners

**DOI:** 10.1002/alz70858_099100

**Published:** 2025-12-25

**Authors:** Jeong Eun Kim, Cristina de Rosa, Joshua D Grill, Jennifer H Lingler

**Affiliations:** ^1^ School of Nursing, University of Pittsburgh, Pittsburgh, PA, USA; ^2^ University of Pittsburgh, Pittsburgh, PA, USA; ^3^ Institute for Memory Impairments and Neurological Disorders, University of California, Irvine, Irvine, CA, USA; ^4^ University of Pittsburgh Alzheimer's Disease Research Center (ADRC), Pittsburgh, PA, USA

## Abstract

**Background:**

Alzheimer's disease and related dementias (ADRD) diagnostics increasingly include biomarker tests. The results of these tests are shared with patients and research participants through methods including mail, in‐person visits, and patient portals. How these methods align with participants' and care partners' (CPs) preferences and needs is largely unknown. Additionally, little is known about how participants and CPs seek further context for their results, such as through educational resources, online searches, or emerging technologies like Artificial Intelligence (AI)‐driven tools. This study aimed to characterize cognitively symptomatic participants' and CPs' preferences for receiving and sharing cognitive health information, and their attitudes toward virtual platforms and AI‐driven systems.

**Method:**

A mixed‐methods analysis was conducted with 50 participants and 36 CPs from a multisite study of patient and family member reactions to biomarker‐informed ADRD diagnoses (PARADE), which recruits participants from various clinical and research settings including CMS Coverage with Evidence Development New IDEAS study. Quantitative questionnaire data were supplemented by focus group discussions (FGDs) to assess preferences for receiving and sharing cognitive health information following ADRD biomarker testing.

**Result:**

Participants (mean age: 72.6 years) and CPs (mean age: 67.6 years) were 54.7% White, 18.6% Black, and 14% Hispanic/Latino. Most were female (60% participants, 61% CPs) and had a bachelor's degree or higher (64% participants, 72.2% CPs). Preferred methods for receiving ADRD‐related test results included paper copies (84% participants, 69.4% CPs) and email (66%, 72.2%). Other preferences were in‐person delivery (50%, 55.6%), telemedicine (46%, 38.9%), and patient portals (38%, 47.2%). Most participants (92%) and CPs (97.2%) were comfortable sharing results with family and friends, and nearly all (98% participants, 97.2% CPs) with healthcare providers. FGDs revealed diverse information‐seeking behaviors and varying attitudes toward AI‐driven tools, from active users to those hesitant or mistrustful. Preferences for virtual formats and group settings to seek cognitive health information often reflected dissatisfaction with current methods and a desire for more accessible, person‐centered systems.

**Conclusion:**

This study highlights diverse preferences for receiving and sharing biomarker testing information. Findings emphasize the need for user‐friendly systems tailored to the evolving needs of individuals navigating ADRD‐related health information.

